# Comparable cellular and humoral immunity upon homologous and heterologous COVID-19 vaccination regimens in kidney transplant recipients

**DOI:** 10.3389/fimmu.2023.1172477

**Published:** 2023-03-31

**Authors:** Nina Körber, Christopher Holzmann-Littig, Gesa Wilkens, Bo-Hung Liao, Maia L. Werz, Louise Platen, Cho-Chin Cheng, Myriam Tellenbach, Verena Kappler, Viktor Lehner, Hrvoje Mijočević, Catharina Christa, Volker Assfalg, Uwe Heemann, Christoph Schmaderer, Ulrike Protzer, Matthias C. Braunisch, Tanja Bauer, Lutz Renders

**Affiliations:** ^1^ Institute of Virology, Helmholtz Zentrum München, Munich, Germany; ^2^ Department of Nephrology, Technical University of Munich, School of Medicine, Klinikum Rechts der Isar, Munich, Germany; ^3^ Technical University of Munich (TUM) Medical Education Center, School of Medicine, Technical University of Munich, Munich, Germany; ^4^ Institute of Virology, Technical University of Munich, School of Medicine, Munich, Germany; ^5^ Department of Surgery, Technical University of Munich, School of Medicine, Klinikum Rechts der Isar, Munich, Germany; ^6^ German Center for Infection Research (DZIF), partner site Munich, Munich, Germany

**Keywords:** COVID-19 vaccination, immunosuppressive therapy, kidney transplant recipients, multiplex Fluorospot, neutralizing antibodies, T-cell responses

## Abstract

**Background:**

Kidney transplant recipients (KTRs) are at high risk for a severe course of coronavirus disease 2019 (COVID-19); thus, effective vaccination is critical. However, the achievement of protective immunogenicity is hampered by immunosuppressive therapies. We assessed cellular and humoral immunity and breakthrough infection rates in KTRs vaccinated with homologous and heterologous COVID-19 vaccination regimens.

**Method:**

We performed a comparative in-depth analysis of severe acute respiratory syndrome coronavirus 2 (SARS-CoV-2)–specific T-cell responses using multiplex Fluorospot assays and SARS-CoV-2-specific neutralizing antibodies (NAbs) between three-times homologously (n = 18) and heterologously (n = 8) vaccinated KTRs.

**Results:**

We detected SARS-CoV-2-reactive T cells in 100% of KTRs upon third vaccination, with comparable frequencies, T-cell expression profiles, and relative interferon γ and interleukin 2 production per single cell between homologously and heterologously vaccinated KTRs. SARS-CoV-2-specific NAb positivity rates were significantly higher in heterologously (87.5%) compared to homologously vaccinated (50.0%) KTRs (*P* < 0.0001), whereas the magnitudes of NAb titers were comparable between both subcohorts after third vaccination. SARS-CoV-2 breakthrough infections occurred in equal numbers in homologously (38.9%) and heterologously (37.5%) vaccinated KTRs with mild-to-moderate courses of COVID-19.

**Conclusion:**

Our data support a more comprehensive assessment of not only humoral but also cellular SARS-CoV-2-specific immunity in KTRs to provide an in-depth understanding about the COVID-19 vaccine–induced immune response in a transplant setting.

## 1 Introduction

The coronavirus disease 2019 (COVID-19) pandemic, with estimated 6.7 million deaths to date (1), has had a significant impact on healthcare systems (2) and the lives of billions of people worldwide (3, 4). Today, a “new normal” (5) is being sought in many countries around the world because the current viral variants seem to trigger severe disease outcomes less frequently (6, 7). Immunocompetent individuals after COVID-19 vaccination develop all effector mechanisms of the adaptive immunity such as severe acute respiratory syndrome coronavirus 2 (SARS-CoV-2)-specific neutralizing antibodies (NAbs) and virus-specific CD4 and CD8 T cells after two vaccine doses, which might be important for controlling infection and preventing severe disease (8–12).

Immunocompromised patients showed themselves to be more vulnerable due to reduced immunological defense (13). First, several immunosuppressed patients are found to have a decreased humoral vaccination response (14–16). Second, other arms of the adaptive immune response are not always considered, and the emerging data so far show an inconsistent picture (17–19). Due to the lower response to the vaccine, more severe courses of COVID-19 and increased mortality are currently found in immunosuppressed patients, even if the overall mortality has been significantly lower as compared to the infection with the delta variant (20–22).

Following the very rapid development of vaccines (23), the question of vaccine efficacy in these special patient groups quickly arose (24).

The aim of this study was to highlight the different pathways of the adaptive immune response after homologous and heterologous COVID-19 vaccinations (two- and threefold) in kidney transplant recipients (KTRs) in particular to contribute to the understanding of not only the less frequently studied T-cell response but also the receptor binding domain (RBD)–specific B-cell response and the serum-neutralizing capacity in this special patient group.

## 2 Materials and methods

### 2.1 Study design

The COVIIMP study (German: “COVID-19-Impfansprechen immunsupprimierter Patient*innen”) is a prospective observational study to examine the COVID-19 immunization success and the clinical course of COVID-19 in immunocompromised patients who received active immunization against SARS-CoV-2 as recommended by the German health authorities. Between 1st April 2021, and 31st August 2022, a total of 513 patients had been enrolled in the COVIIMP study. Participants were immunocompromised due to immunosuppressive medication after kidney transplantation, a rheumatologic disease under immunosuppressive therapy or maintenance hemodialysis (MHD). All patients were included before their fourth vaccination.

The study was performed at the University Hospital rechts der Isar in Munich and collaborating outpatient dialysis centers. All participating patients provided written informed consent. The study adheres to the Declaration of Helsinki. It was approved by the Medical Ethics Committee of the Klinikum Rechts der Isar of the Technical University of Munich (approval number 163/21 S-SR, 19th March 2021) and registered at the Paul Ehrlich Institute (NIS592) (25).

### 2.2 Study population

Of 513 patients enrolled in the COVIIMP study, a total of 26 SARS-CoV-2-naïve KTRs (median age 57.0 years (49.5–62.9 years), 34.6% women) (**Table 1**) were enrolled in this part of the study to examine SARS-CoV-2-specific humoral and cellular immunity after the application of homologous and heterologous COVID-19 vaccination regimens. For homologous vaccination (n = 18), KTRs received two and later on a third dose of either BNT162b2 mRNA (Comirnaty^®^, BioNTech-Pfizer) or mRNA-1273 (Spikevax^®^, Moderna Biotech) vaccines. For heterologous vaccination (n = 8), one dose of ChAdOx1 nCoV-19 (Vaxzevria^®^, AstraZeneca) was followed by one or two doses of one of the two mRNA vaccines. Patients with previous SARS-CoV-2 infections were identified by PCR or at least one positive serological SARS-CoV-2 nucleocapsid-specific Immunoglobulin G (IgG) assay result (12, 26) and subsequently excluded from the study.

**Table 1:** Patient characteristics.

		Vaccination scheme		
Variable	KTR (n = 26)	Homologous (n = 18)	Heterologous (n = 8)	*P-*value^A^
Vaccine (first/second/third dose(s)		mRNA/mRNA/mRNA	vector/mRNA/mRNA	
Age (years)	57.0 (49.5-62.9)	61.6 (51.4–69.8)	53.0 (47.2–56.9)	0.102
Sex: female	9 (34.6%)	7 (38.9%)	2 (25.0%)	0.667
Underlying disease				
Congenital or cystic renal disease	6 (23.1%)	4 (22.2%)	2 (25.0%)	>0.999
Glomerulopathy	8 (30.8%)	4 (22.2%)	4 (50.0%)	0.197
Hypertensive nephropathy	2 (7.7%)	2 (11.1%)	0 (0.0%)	>0.999
Nephropathy of unknown origin	6 (23.1%)	6 (33.3%)	0 (0.0%)	0.132
Reflux nephropathy	1 (3.8%)	0 (0.0%)	1 (12.5%)	0.308
Other	3 (11.5%)	2 (11.1%)	1 (12.5%)	>0.999
Charlson Comorbidity Index	2.5 (2.0-4.75)	4.0 (2.0–5.0)	2.0 (1.8–3.3)	0.198
Cardiovascular comorbidity**	7 (26.9%)	6 (33.3%)	1 (12.5%)	0.375
Diabetes mellitus	7 (26.9%)	4 (22.2%)	3 (37.5%)	0.635
eGFR (ml/min)	41.0 (34.1–58.0)	40.5 (34.0–55.8)	57.0 (40.5–60.0)	0.380
CRP (mg/dl)	0.3 (0.1–0.5)	0.2 (0.1–0.5)	0.4 (0.3–0.6)	0.246
WBC (G/L)	6.8 (6.0–7.8)	6.8 (5.6–8.0)	6.6 (6.4–7.7)	0.664
Lymphocytes	25.5 (20.25-29.93)	25.0 (20.5–29.5)	29.0 (19.0–31.9)	0.860
Time after (last) transplantation (years)	5.1 (1.2-9.0)	5.3 (0.9–9.0)	5.1 (2.6–7.3)	0.683
Time between second and third vaccination (days)		188 (174–202)	158 (151–167)	0.043
Last transplant—living donor	11 (42.3%)	6 (33.3%)	5 (62.5%)	0.218
Number of transplantations				
1	23 (88.5%)	17 (94.4%)	6 (75.0%)	0.215
2	2 (7.7%)	0 (0.0%)	2 (25.0%)	0.086
3	1 (3.8%)	1 (5.6%)	0 (0.0%)	>0.999
History of CMV	12 (46.2%)	10 (55.6%)	2 (25.0%)	0.216
History of BK virus	7 (26.9%)	5 (27.8%)	2 (25.0%)	>0.999
History of biopsy-proven rejection	11 (42.3%)	9 (50.0%)	2 (25.0%)	0.395
Immunosuppressive medication				
Mono therapy	3 (11.5%)	3 (16.7%)	0 (0.0%)	0.529
Dual therapy	3 (11.5%)	1 (5.6%)	2 (25.0%)	0.215
Triple therapy	20 (76.9%)	14 (77.8%)	6 (75.0%)	>0.999
Tacrolimus	22 (84.6%)	15 (83.3%)	7 (87.5%)	>0.999
MMF	22 (84.6%)	14 (77.8%)	8 (100.0%)	0.277
Corticosteroids	20 (76.9%)	14 (77.8%)	6 (75.0%)	>0.999
mTOR	2 (7.7%)	2 (11.1%)	0 (0.0%)	>0.999
Cyclosporine	2 (7.7%)	1 (5.6%)	1 (12.5%)	0.529
Other	0 (0.0%)	0 (0.0%)	0 (0.0%)	>0.999

**Cardiovascular comorbidity: myocardial infarction, congestive heart failure, peripheral arterial disease, cerebrovascular disease; CMV, cytomegalovirus; eGFR, estimated glomerular filtration rate; KTR, kidney transplant recipient; MMF, mycophenolate mofetil. mTOR, mammalian target of rapamycin; WBC, white blood cell count. ^A^ Comparing homologously and heterologously COVID-19 vaccinated KTRs with Mann–Whitney U test or Fisher’s exact test. Values presented as median (IQR) and n (%).

### 2.3 Sample collection

Blood was drawn for analysis in median 21 days (12–46 d) (after second vaccination) and in median 17 days (13–36d) (after third vaccination) after the second and third vaccination, respectively (Additional file 1: **Figure S1**).

### 2.4 Severe acute respiratory syndrome coronavirus 2-nucleocapsid-specific IgG antibodies and surrogate neutralization assay

Serological analyses were performed on the iFlash 1800 platform (Yhlo Biotechnology) using a surrogate paramagnetic particle chemiluminescence immunoassay (CLIA). Patients’ sera were screened for previous SARS-CoV-2 infections *via* the detection of nucleocapsid-specific IgG (anti-N IgG), and a reactive result (anti-N IgG ≥10 AU/ml) led to exclusion from the study. SARS-CoV-2-specific NAb directed against the RBD within the S1 subunit of SARS-CoV-2 may prevent the infection of host cells *via* the viral entry receptor angiotensin-converting enzyme II (ACE2) (27). Surrogate neutralization was quantified using the commercial iFlash-2019-nCoV NAb kit. SARS-CoV-2-specific NAb in patients’ sera form a complex with SARS-CoV-2 RBD antigen-coated paramagnetic microparticles. Acridinium-ester-labeled ACE2 conjugates competitively bind the remaining RBD. This reaction mixture creates high relative light units (28) and inversely correlates to the amount of SARS-CoV-2-specific NAb, which is calculated in AU/ml (29, 30). Values ≥ 10 AU/ml were considered seropositive. According to the manufacturer’s specifications, the lower and upper limit of quantification (LLOQ/ULOQ) is 4 and 800 AU/ml, respectively. Values exceeding ULOQ were entered as 801 AU/ml in the statistical analysis. A conversion factor allows for the adaptation to the WHO standard (NIBSC code 20/136) (AU/ml × 2.4 = IU/m/l).

### 2.5 Severe acute respiratory syndrome coronavirus 2 virus

SARS-CoV-2 Omicron sublineage BA.5 (GISAID EPI_ISL_15942298.) was isolated from the nasopharyngeal swabs of COVID-19-infected patients. Vero-E6 cells (tested for mycoplasma contamination) were infected with the virus and incubated in a cell culture medium (Dulbecco’s modified Eagle’s medium supplemented with 10% fetal calf serum, 1% glutamine, 1% penicillin-streptomycin, 1% non-essential amino acid, and 1% sodium pyruvate). The cell supernatant was then collected after the virus-induced cytopathic effect appeared. After discarding the cell debris by centrifugation, the supernatant with virus particles was collected and stored at -80°C as virus stock. Strain identity was confirmed by next-generation sequencing, and the titer was determined with the plaque assay.

### 2.6 Severe acute respiratory syndrome coronavirus 2 infection-neutralization assay

Before analysis, patient serum samples were maintained at -80°C for storage and then thawed and held at 4°C for 1/day. To assess neutralizing capabilities, sera were diluted 20- to 2,560-fold using a twofold serial dilution and incubated with SARS-CoV-2 Omicron sublineage BA.5 at a predetermined multiplicity of infection (MOI) of 0.03 (450 PFU/15,000 cells/well) at 37°C for 1 h. After 1 h of incubation, the inoculum was transferred to Vero-E6 cells for an additional hour of incubation at 37°C with 5% CO_2_. The inoculum was replaced with cell culture media (Dulbecco’s modified Eagle’s medium with 10% fetal calf serum, 1% glutamine, 1% penicillin–streptomycin, 1% non-essential amino acid, and 1% sodium pyruvate). After 24 h, the infection was terminated, and an in-cell ELISA assay was performed. In-cell ELISA was initiated by the fixation of cells with 4% paraformaldehyde, the permeabilization of cell membranes with 0.5% saponin buffer, and blocking with 10% goat serum. After blocking, cells were incubated with an anti-SARS-CoV-2 nucleocapsid primary antibody (40143-T62, Sino Biological, Beijing, China) followed by a goat anti-rabbit IgG2a-HRP secondary antibody (EMD Millipore/#12-355, Shanghai, China). By adding a substrate tetramethylbenzidine buffer (TMB), the HRP signals were converted to colorimetric signals and read at 450 nm by Infinite^®^ 200 PRO (Tecan Trading AG, Switzerland). The IC50 values, which reflect the dilution factor at which 50% infection inhibition was achieved for each blood sample, were then determined using non-linear regression. The neutralizing capabilities were categorized as under detection if the IC50 values were less than 20-fold dilution and as >2,560 if the IC50 values were greater than 2560-fold dilution.

### 2.7 Isolation and cryopreservation of peripheral blood mononuclear cells

Blood from study participants was drawn with the Vacutainer CPT™ system into sodium citrate CPT tubes (Becton Dickinson Biosciences), and the tubes were mixed five times before storing them upright at room temperature. Within 2 h of blood collection, CPT tubes were centrifuged in a horizontal rotor (swing-out head) (1,800 g, 20 min, RT). Next, plasma was removed and peripheral blood mononuclear cells (PBMCs) were transferred to 50 ml polystyrene Falcon tubes and mixed with 10 ml of PBS by gently inverting the tubes five times and filled up to 45 ml with PBS. PBMCs were centrifuged (300 g, 10 min, RT) twice in PBS. Tuerk solution staining (Morphisto GmbH) was used for cell counting under the microscope. PBMCs were cryopreserved per vial in 1.8 ml cryotubes (Thermo Scientific) at a concentration of 1 × 10^7^ PBMCs per 1 ml of freezing medium/[fetal calf serum (FCS) (Life Technologies)], supplemented with 10% DMSO (Sigma-Aldrich), using a freezing container (Thermo Scientific) and stored at −80°C. After 24 h, PBMCs were stored in the vapor phase of a liquid nitrogen tank until further use.

### 2.8 Thawing and resting of peripheral blood mononuclear cells

PBMCs were thawed and rested as described previously (31). Cell counting was performed on an ImmunoSpot Ultimate UV Image analyzer (CTL Europe GmbH) as described previously (12).

### 2.9 Dual-color interferon γ/interleukin 2 Fluorospot assay

Human IFN-γ/IL-2 dual-color Fluorospot assays (CTL Europe GmbH) were performed according to the manufacturer’s instructions. The plates were activated by adding 70% ethanol for less than 1 min 1 day before the Fluorospot assays were performed, followed by a washing step and the addition of IFN-γ/IL-2 capture antibodies overnight, respectively. After decanting the plate, PBMCs were placed at a concentration of 2 × 10^5^/well in a final volume of 200 μl/well. PBMCs were stimulated for 22 h with 1 μg/ml of overlapping peptide pools (15mers overlapping by 11 aa) of the SARS-CoV-2 spike protein (PepMix™ SARS-CoV-2 (PM-WCPV-S; JPT Peptide Technologies), consisting of two peptide pools, i.e., S1 and S2 with 158 and 157 peptides, respectively. As an antigen-specific positive control, we used a CEF pool of in total 32 peptides derived from cytomegalovirus (5 peptides), Epstein–Barr virus (15 peptides), and influenza virus (flu) (12 peptides) proteins (National Institute for Biological Standards and Control). After the stimulation period, the plates were washed and 80 μl of either anti-human IFN-γ (FITC) or anti-human IL-2 (Hapten2) detection antibody solution was added for additional 2 h. For the visualization of secreted cytokines, plates were washed and a tertiary solution including either anti-FITC Alexa Fluor^®^ 488 (visualizes IFN-γ) or anti-Hapten2 CTL-Red™ (visualizes IL-2) was added for 1 h. The staining procedure was stopped by washing the plate. After drying the plates for 24 h on paper towels, Fluorospot plates were scanned using an automated reader system (ImmunoSpot Ultimate UV Image analyzer/ImmunoSpot 7.0.35.0 Professional DC Software, CTL Europe GmbH). The counting of spot-forming cells (SFCs) on Fluorospot plates was performed by adjusting the sensitivity, background balance, and gates for the spot size using the CTL software. Counting was performed in compliance with the guidelines (32) for the automated evaluation of ELISpot assays. All counts were reviewed and certified by a second person in a rigorous quality control process. The final results are represented as SFCs per 1 × 10^6^ PBMCs. Positive reactivity to experimental stimulatory agents was given when the spot count in antigen-stimulated wells was greater than twice the spot count in unstimulated (background) wells. Wells that did not meet the criteria for a positive response were set at 0 SFC/well.

For the analysis of the cytokine production per single SFC, we determined the mean intensity, which is defined as the arithmetic mean of the intensity function values of the counted image for the spot pixels and the spot size using the CTL software. The values for the cytokine production per SFC were calculated by the product of “mean intensity” * “spot size,” and, as such, represent the total amount of “fluorescence” of a spot. The unit is %max * µm^2^.

### 2.10 Human IgG severe acute respiratory syndrome coronavirus 2 receptor binding domain ELISpot

The human IgG SARS-CoV-2 RBD ELISpot^PLUS^ kit (Mabtech AB) was used for the enumeration of memory B cells secreting IgG antibodies specific for the SARS-CoV-2 RBD. Prior to the ELISpot assay, thawed PBMCs were cultured for 4 days in a serum-free CTL-Test™ B Medium (CTL Europe GmbH) containing R848 (1 µg/ml) and recombinant IL-2 (10 ng/ml) for the *in vitro* prestimulation of memory B cells as recommended by the manufacturer (Mabtech AB). The ELISpot assay was performed in triplicates according to the manufacturer’s instructions using 5 × 10^5^ preactivated cells per well and a stimulation time of 22 h. The counts of RBD-specific IgG-secreting B cells exceeding ULOQ were set to 1,000 SFC per 1 × 10^6^ PBMC to enable statistical analysis.

### 2.11 Statistical analysis

Categorical variables are presented as frequencies and percentages. Continuous variables are expressed as median and interquartile range (IQR). Group differences were tested with the Fisher’s exact test, and the Mann–Whitney U test was used for continuous variables. Paired samples were examined with the Wilcoxon test. All tests were conducted two-sided, and *P* < 0.05 was considered significant. Statistical analysis was performed using R version 4.0.2 (R Foundation for statistical Computing, Vienna, Austria) and Graph Pad Prism (Version 9.4.1).

### 2.12 Study approval

The study adheres to the Declaration of Helsinki. It was approved by the Medical Ethics Committee of the Klinikum Rechts der Isar of the Technical University Munich (approval number 163/21 S-SR, 19th March 2021) and registered at the Paul Ehrlich Institute (NIS592). Written informed consent was received from participants prior to inclusion in the study.

## 3 Results

### 3.1 Patient characteristics

**Figure 1:**
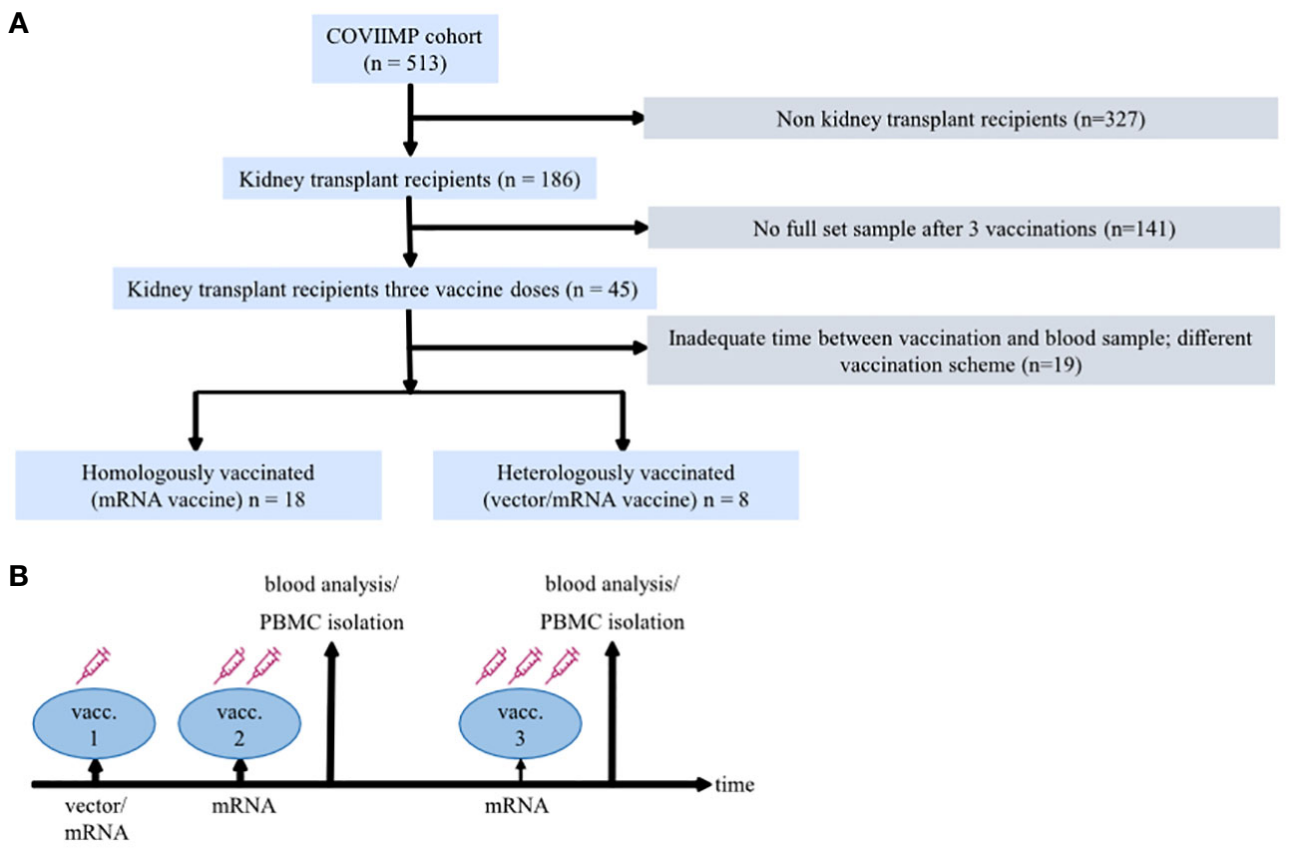
Study design. **(A)** Diagram describing the flow of patient enrollment and exclusion. **(B)** A schematic of the study design illustrating applied coronavirus disease 2019 (COVID-19) vaccination regimens, doses, and the time of blood sampling.

A total of 26 KTRs could be included in the study (**Figure 1A**). Of these, 18 patients were homologously COVID-19 vaccinated (hoVac) (first/second/third vaccine dose(s): mRNA/mRNA/mRNA vaccine), and 8 patients were heterologously vaccinated (heVac) (first/second/third vaccine dose(s): vector/mRNA/mRNA vaccine) (**Figure 1B**). All participants with heterologous vaccination received the dose of ChAdOx1 nCoV-19 (Vaxzevria^®^, AstraZeneca) before the mRNA vaccines. The median age of the patients was 57.0 years, and approximately one-third were women. The median Charlson Comorbidity Index (33) was 2.5, and the median estimated glomerular filtration rate (eGFR) was 41.0 ml/min. About three-fourths of the patients had triple immunosuppression. There were no significant differences between the two groups (hoVac vs. heVac) with respect to age, sex, underlying disease, the Charlson Comorbidity Index, eGFR, CRP, blood count, time since transplantation, CMV and BK viremia, and the type of immunosuppression (**Table 1**). Vaccination reaction symptoms were reported by 14 (77.8%) of the participants in the hoVac and 8 (100%) of participants in the heVac group. None of the patients experienced severe side effects.

### 3.2 Different severe acute respiratory syndrome coronavirus 2–specific T-cell kinetics between homologously vaccinated compared to heterologously vaccinated kidney transplant recipients after second but not after third vaccine dose

SARS-CoV-2-specific T-cell reactivity (i.e. response to spike peptide pool S1 and S2 stimulation) was determined by IFN-γ/IL-2 Fluorospot assays after second and third vaccination.

Overall spike-reactive T-cell response rates tended to be higher (*P* = 0.071) in heVac compared to hoVac KTRs after second vaccination (100% (8/8) vs. 77.8% (14/18)), but were equally high (100% response rate in both cohorts) after the third vaccine dose (**Table 2**).

**Table 2:** Ratios of severe acute respiratory syndrome coronavirus 2 (SARS-CoV-2)–reactive T-cell- and SARS-CoV-2-specific neutralizing antibody (NAb)–positivity in homologously and heterologously COVID-19 vaccinated kidney transplant recipients (KTRs).

		Vaccination scheme		
	KTR(n = 26)	Homologous, mRNA only (n = 18)	Heterologous, vector/mRNA (n = 8)	*P-*value^A^
After second vaccination				
S1-reactive T cells	19 (73.1%)	11 (61.1%)	8 (100%)	0.062
S2-reactive T cells	21 (80.8%)	13 (72.2%)	8 (100%)	0.281
Total spike-reactive T cells^B^	22 (84.6%)	14 (77.8%)	8 (100%)	0.071
After third vaccination				
S1-reactive T cells	25 (96.2%)	17 (94.4%)	8 (100%)	>0.999
S2-reactive T cells	26 (100%)	18 (100%)	8 (100%)	>0.999
Total spike-reactive T cells^B^	26 (100%)	18 (100%)	8 (100%)	>0.999

KTR, kidney transplant recipients. ^A^Comparing homologously and heterologously vaccinated KTRs with Fisher’s exact test. ^B^Total spike-reactive T cells = sum of S1- and S2-reactive T cells. Values are presented as n (%).

In detail, we detected spike S1- and S2-reactive IFN-γ and/or IL-2 secreting cells in 61.1% (11/18) and 72.2% (13/18) of hoVac KTRs after second COVID-19 vaccination (**Table 2**). After third vaccination, all except one hoVac KTR showed spike S1-reactive T cells (17/18, 94.4%), whereas S2-reactive T cells were detectable in all hoVac KTRs (18/18, 100%) (**Table 2**). Noteworthy, all (8/8) heVac KTRs showed spike S1- and S2-reactive IFN-γ and/or IL-2 secreting cells already upon second and subsequently also upon third vaccination (**Table 2**).

**Figure 2:**
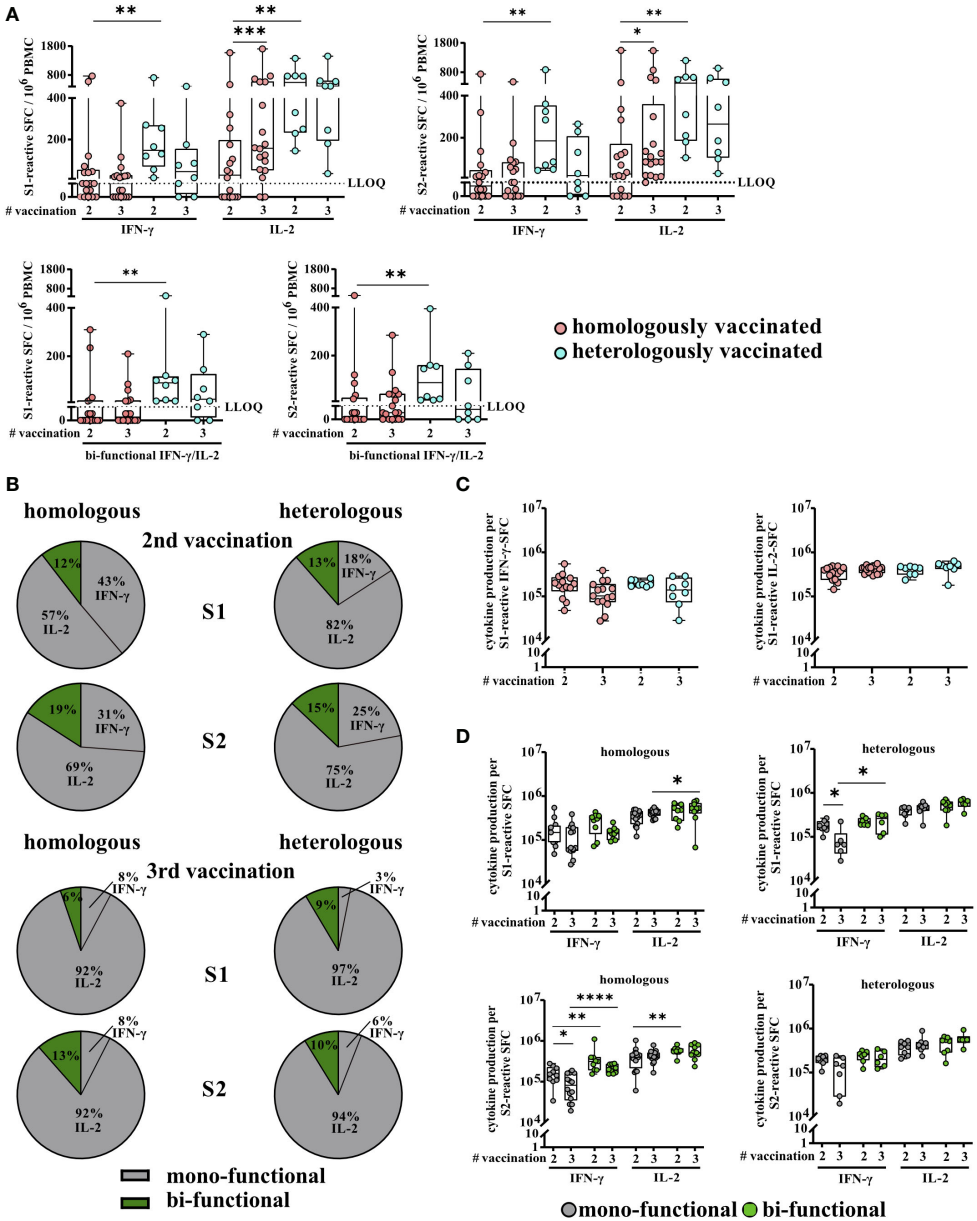
Severe acute respiratory syndrome coronavirus 2 (SARS)-CoV-2-specific T-cell responses of homologously and heterologously vaccinated kidney transplant recipients (KTRs) after second and third COVID-19 vaccination. **(A)** Numbers of spike (S1 or S2)-reactive interferon γ (IFN-γ), interleukin 2 (IL-2), and bifunctional IFN-γ/IL-2 cytokine-secreting cells of homologously (hoVac) (pink) or heterologously (turquoise) (heVac) vaccinated KTRs after two or three doses of COVID-19 vaccines depicted as spot-forming cells (SFCs) per 10^6^ PBMC. **(B)** Cytokine expression profile of spike (S1 or S2)-reactive T cells of hoVac or heVac KTRs after two or three doses of COVID-19 vaccines. Pie charts show the proportions of mono- (gray) and bifunctional (green) cells, respectively. **(C)** Comparison of IFN-γ and IL-2 production per spike S1-reactive cytokine-secreting cells of hoVac or heVac KTRs after two or three doses of COVID-19 vaccines. Cytokine production per cell was determined by the parameters’ spot size and spot intensity as described in methods. **(D)** Comparison of cytokine production per spike (S1 or S2)-reactive mono- and bifunctional cytokine-secreting cells of hoVac or heVac KTRs after two or three doses of COVID-19 vaccines. Cytokine production per cell was determined as described in **(C)** and methods. Statistical analyses by two-sided Mann–Whitney U and Wilcoxon signed-rank tests and Fisher’s exact t test. Solely significant differences are indicated with an asterisk in the graphs. *P < 0.05; **P < 0.01; ***p < 0.001; ****P < 0.0001. LLOQ, lower limit of quantification.

Aside from overall higher T-cell response rates, heVac KTRs also showed significantly higher (individual) numbers of spike S1- and S2-reactive IFN-γ, IL-2, and bi-functional IFN-γ/IL-2 secreting cells (S1, IFN-γ: *P* = 0.0042, IL-2: *P* = 0.0019, IFN-γ/IL-2: *P* = 0.0019 and S2, IFN-γ: *P* = 0.0017, IL-2: P = 0.0039, IFN-γ/IL-2: *P* = 0.0031, respectively) after receiving two doses of COVID-19 vaccines (**Figure 2A**). After a third vaccine dose, these significant differences dispersed and numbers of spike S1- and S2-reactive IFN-γ, IL-2, and bi-functional IFN-γ/IL-2 secreting cells were comparable between both sub-cohorts (**Figure 2A**). Within the cohort of hoVac KTRs, we observed a further significant increase in numbers of spike S1- and S2-reactive IL-2 secreting cells (*P* = 0.0003 and *P* = 0.0277, respectively) after third vaccination, whereas detectable numbers of IFN-γ and bifunctional IFN-γ/IL-2 secreting cells remained stable (**Figure 2A**). We observed no significant changes of spike-reactive T-cell numbers upon third vaccination for heVac KTRs (**Figure 2A**).

The proportions of SARS-CoV-2-reactive mono- (antigen-specific cells secreting either IFN-γ or IL-2) and bifunctional (antigen-specific cells secreting IFN-γ and IL-2 simultaneously) cells in the spike S1- and S2-specific T-cell response were comparable between hoVac and heVac KTRs at both time points (second vaccination: S1: 88% and 87% mono- and 12% and 13% bifunctional cells; S2: 81% and 85% mono- and 19% and 15% bifunctional cells, respectively; third vaccination: S1: 94% and 91% mono- and 6% and 9% bifunctional cells; S2: 87% and 90% mono- and 13% and 10% bifunctional cells, respectively) (**Figure 2B**). Overall, in both cohorts, monofunctional spike S1- and S2-reactive T cells showed a significant dominance of IL-2 secretion with only low proportions of monofunctional IFN-γ-secreting cells at both time points (*P* < 0.0001, respectively; except for S1-reactive cells within the hoVac KTR) (**Figure 2B**). This IL-2 dominance was even more pronounced after the third vaccination with 92% spike S1- and S2-reactive monofunctional IL-2-secreting cells in hoVac and 97% and 94% spike S1- and S2-reactive monofunctional IL-2-secreting cells in heVac KTRs (**Figure 2B**). Noteworthy, in both subcohorts, the numbers of bifunctional spike S1- and S2-reactive T cells tend to be lower after third vaccination accompanied by an increase of monofunctional cells (**Figure 2B**).

As a further parameter of T-cell activity/T-cell response, we determined the cytokine production of SARS-CoV-2-specific T cells per single cell and observed no significant differences for spike S1- and S2-reactive IFN-γ- and IL-2-secreting cells between hoVac and heVac KTRs after second and third COVID-19 vaccination (**Figure 2C** and Additional file 2: **Figure S2**). This was also the case for the comparison of the cytokine production of mono- and bifunctional spike S1- and S2-reactive T cells between both cohorts at both time points. However, in both cohorts, bifunctional cells showed a tendency, partially significant, of a higher cytokine production per SFC compared to monofunctional cells (**Figure 2D**). Furthermore, we observed a significantly higher cytokine production of spike S1- and S2-reactive IL-2 compared to IFN-γ-secreting cells in both cohorts and at both time points (**Figure 2D**).

In summary, heVac KTRs showed higher overall T-cell response rates and significantly higher numbers of SARS-CoV-2-specific T cells after two but not after three COVID-19 vaccinations. In contrast, the T-cell cytokine expression profile and the cytokine production per spike S1- and S2-reactive T cells were comparable between both subcohorts at both time points.

### 3.3 Higher lymphocyte counts in kidney transplant recipients who did not develop severe acute respiratory syndrome coronavirus 2–specific T-cell responses after second vaccination

We detected significantly higher lymphocyte counts in KTRs who did not develop SARS-CoV-2-specific T-cell responses after second vaccination (37.0% vs. 24.0%, *P* = 0.013) (**Table 3**). There were no significant differences between the two groups (SARS-CoV-2 T-cell positive and -negative KTRs) with respect to age, sex, Charlson Comorbidity Index, eGFR, CRP, WBC, CMV and BK viremia, and the type of immunosuppression (**Table 3**). Since all of the tested KTRs developed SARS-CoV-2-specific T-cell responses after third vaccination, we did not perform previously described statistical analyses for this time point.

**Table 3:** Characteristics of KTRs stratified by SARS-CoV-2 T-cell positivity after second vaccination.

Variable	T-cell-negative KTR (n = 4)	T-cell-positive KTR (n = 22)	*P-* value^A^
Age (years)	75.9 (63.9–80.7)	55.3 (49.6–61.9)	0.096
Sex: female	0 (0.0%)	9 (40.9%)	0.263
Charlson Comorbidity Index	6.0 (4.0–7.5)	2.0 (2.0–4.0)	0.119
Diabetes mellitus	2 (50.0%)	5 (22.7%)	0.287
eGFR (ml/min)	35.6 (32.1–40.8)	42.0 (38.5–60.3)	0.223
CRP (mg/dl)	0.2 (0.2–0.4)	0.3 (0.2–0.6)	0.622
WBC (G/L)	6.3 (4.9–7.9)	6.8 (6.4–7.8)	0.682
Lymphocytes	37.0 (36.0–38.0)	24.0 (18.5–29.0)	0.013
History of CMV	1 (25.0%)	11 (50.0%)	0.598
History of BK virus	0 (0.0%)	7 (31.8%)	0.546
Immunosuppressive medication			
Mono therapy	0 (0.0%)	3 (13.6%)	>0.999
Dual therapy	1 (25.0%)	2 (9.1%)	0.401
Triple therapy	3 (75.0%)	17 (77.3%)	>0.999
Tacrolimus	3 (75.0%)	19 (86.4%)	0.511
MMF	4 (100%)	19 (86.4%)	>0.999
Corticosteroids	3 (75.0%)	17 (77.3%)	>0.999
mTOR	0 (0.0%)	2 (9.1%)	>0.999
Cyclosporine	0 (0.0%)	2 (9.1%)	>0.999
Other	1 (25.0%)	0 (0.0%)	0.154

CMV, cytomegalovirus; CRP, C-reactive protein; eGFR, estimated glomerular filtration rate; KTR, kidney transplant recipient; MMF, mycophenolate mofetil; mTOR, mammalian target of rapamycin; WBC, white blood cell count. ^A^Comparing T-cell negative and T-cell positive KTRs with Mann–Whitney U test or Fisher’s exact test. Values are presented as median (IQR) and n (%).

### 3.4 Significantly higher severe acute respiratory syndrome coronavirus 2–specific neutralizing antibody–positivity rates but comparable neutralizing antibody titers in heterologously vaccinated compared to homologously vaccinated kidney transplant recipients

**Figure 3:**
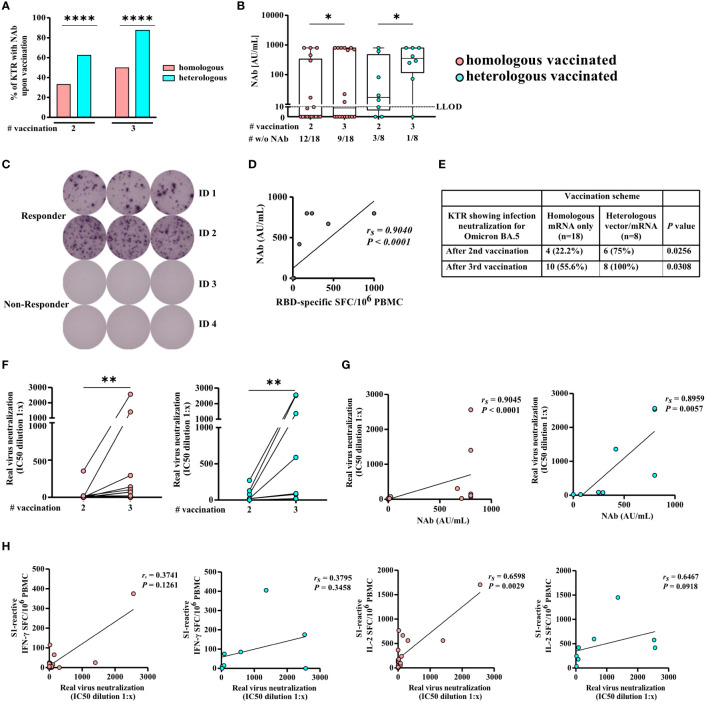
SARS-CoV-2-specific NAb and serum neutralization capacity for Omicron BA.5 of homologously and heterologously COVID-19 vaccinated KTRs. Illustrated is **(A)** the percentage and **(B)** NAb titers of homologously (hoVac) and heterologously (heVac) vaccinated KTRs who developed SARS-CoV-2-specific NAbs after two or three doses of COVID-19 vaccines determined by a surrogate neutralization assay and given in AU/ml. **(C)** Exemplarily staining of RBD-specific memory B cells of hoVac KTRs with (responder) or without (non-responder) spike-specific IgG NAbs after three doses of COVID-19 vaccines. **(D)** Correlation of SARS-CoV-2-specific NAbs and the numbers of RBD-specific memory B cells of hoVac and heVac KTRs after three doses of COVID-19 vaccines. **(E)** Percentage of hoVac and heVac KTRs showing infection neutralization activity for Omicron BA.5 after second and third vaccination. **(F)** Changes in serum neutralization capacities for Omicron BA.5 after second and third COVID-19 vaccination. Inhibitory concentration (IC50) dilution values are given. Dots indicate the measurement of an individual patient with lines connecting individual values after second and third vaccination. **(G, H)** Spearman correlation of serum neutralization capacities for Omicron BA.5 and the NAb titer **(G)** and the numbers of spike S1-specific IFN-γ- and IL-2-secreting cells depicted as spot-forming cells (SFCs) per 10^6^ PBMCs **(H)** after third vaccination of hoVac and heVac KTRs. Statistical analyses by the two-sided Mann–Whitney U test, Wilcoxon signed-rank test, Fisher’s exact t test, and Spearman correlation and linear regression analysis. Solely significant differences are indicated with an asterisk in the graphs. *P < 0.05; **P < 0.01; ****P < 0.001. LLOD = lower limit of detection. *r_s_* denotes the Spearman correlation coefficient.

SARS-CoV-2-specific NAb-positivity rates were significantly higher in heVac [62.5% (5/8) and 87.5% (7/8)] compared to hoVac [33.3% (6/18) and 50.0% (9/18)] KTRs upon second and third vaccination (*P* < 0.0001), respectively (**Figure 3A**), whereas we observed no significant differences while comparing SARS-CoV-2-specific NAb titers between hoVac and heVac KTRs after second and third vaccination (**Figure 3B**). Upon third vaccination, SARS-CoV-2-specific NAb titers significantly increased in both cohorts (*P* = 0.0313, respectively) (**Figure 3B**).

### 3.5 Severe acute respiratory syndrome coronavirus 2–specific neutralizing antibody–negative kidney transplant recipients had significantly lower glomerular filtration rates and were without exception under mycophenolate mofetil therapy

We observed significantly lower eGFR in KTRs who did not develop SARS-CoV-2-specific NAbs after second and third vaccination (*P* = 0.046, respectively) (**Table 4**). Moreover, NAb-negative KTRs after second vaccination were without exception under mycophenolate mofetil (MMF) therapy (100%, *P* = 0.022) and showed a tendency of being more frequent under corticosteroid and triple therapy compared to NAb-positive KTRs (**Table 4**). There were no significant differences between the two groups (NAb-negative and NAb-positive KTRs) with respect to age, sex, Charlson Comorbidity Index, CRP, blood count, CMV, and BK viremia after second and third vaccination (**Table 4**).

**Table 4:** Characteristics of KTRs stratified by SARS-CoV-2-specific NAb-positivity after second (left) and third (right) vaccination.

Variable	KTRs with NAbs < 10 AU/ml (n = 15)	KTRs with NAbs > 10 AU/ml (n = 11)	*P-* value^A^	KTRs with NAbs < 10 AU/ml (n = 10)	KTRs with NAbs > 10 AU/ml (n = 16)	*P-* value^A^
Age (years)	52.3 (42.7–72.1)	59.1 (55.1–62.1)	0.540	54.7 (42.2–72.5)	57.0 (52.5–62.1)	0.979
Sex: female	4 (26.7%)	5 (45.5%)	0.699	2 (20.0%)	7 (43.8%)	0.399
Charlson Comorbidity Index	2.0 (2.0–5.0)	4.0 (2.0–4.0)	0.929	2.0 (2.0–5.0)	3.5 (2.0–4.0)	0.967
Diabetes mellitus	3 (20.0%)	3 (27.3%)	>0.999	2 (20.0%)	4 (25.0%)	>0.999
eGFR (ml/min)	40.0 (32.0–46.5)	57.0 (42.0–61.8)	0.046	38.5 (28.0–41.0)	57.0 (40.0–61.5)	0.046
CRP (mg/dl)	0.3 (0.2–0.6)	0.3 (0.1–0.5)	>0.999	0.3 (0.2–0.7)	0.3 (0.2–0.5)	0.711
WBC (G/L)	6.9 (5.2–7.8)	6.8 (6.6–7.8)	0.546	6.1 (5.2–7.7)	6.8 (6.5–7.8)	0.412
Lymphocytes	29.0 (22.0–33.0)	24.0 (16.0–26.0)	0.215	29.0 (26.0–31.3)	23.5 (20.3–28.8)	0.304
History of CMV	7 (46.7%)	5 (45.5%)	>0.999	4 (40.0%)	8 (50.0%)	0.702
History of BK virus	4 (26.7%)	3 (27.3%)	>0.999	2 (20.0%)	5 (31.3%)	0.668
Immunosuppressive medication						
Mono therapy	0 (0.0%)	3 (27.3%)	0.064	0 (0.0%)	3 (18.8%)	0.262
Dual therapy	1 (6.7%)	2 (18.2%)	0.556	1 (10.0%)	2 (12.5%)	>0.999
Triple therapy	14 (93.3%)	6 (54.5%)	0.054	9 (90.0%)	11 (68.9%)	0.352
Tacrolimus	13 (86.7%)	9 (81.8%)	>0.999	8 (80.0%)	14 (87.5%)	0.625
MMF	15 (100%)	7 (63.6%)	0.022	10 (100%)	12 (75.0%)	0.136
Corticosteroids	14 (93.3%)	6 (54.5%)	0.054	9 (90.0%)	11 (68.8%)	0.352
mTOR	0 (0.0%)	2 (18.2%)	0.169	0 (0.0%)	2 (12.5%)	0.508
Cyclosporine	1 (6.7%)	1 (9.1%)	>0.999	1 (10.0%)	1 (6.3%)	>0.999
Other	1 (6.7%)	0 (0.0%)	>0.999	1 (10.0%)	0 (0.0%)	0.385

CMV, cytomegalovirus; CRP, C-reactive protein; eGFR, estimated glomerular filtration rate; KTR, kidney transplant recipient; MMF, mycophenolate mofetil; mTOR, mammalian target of rapamycin; WBC, white blood cell count. ^A^Comparing KTRs with SARS-CoV-2-specific NAbs < 10 AU/ml and NAbs > 10 AU/ml with Mann–Whitney U test or Fisher’s exact test. Values are presented as median (IQR) and n (%).

### 3.6 Receptor binding domain–specific memory B cells in the absence of measurable levels of serum severe acute respiratory syndrome coronavirus 2–specific neutralizing antibodies

Vaccine-induced SARS-CoV-2-specific memory B cells can be reactivated upon reexposure to antigen and might help to prevent a severe course of COVID-19. We performed B-cell ELISpot assays to evaluate whether KTRs who did not develop detectable SARS-CoV-2-specific NAbs upon third COVID-19 vaccination established/still have RBD-specific memory B cells (see exemplary ELISpot wells in **Figure 3C**). We detected RBD-specific IgG-secreting B cells in 36.4% (4/11) (median 47, range 14–436 RBD-specific IgG-secreting B cells/10^6^ PBMC) of KTR-tested negative for SARS-CoV-2-specific NAbs. In a control group of KTRs who tested positive for SARS-CoV-2-specific NAbs, we detected RBD-specific IgG-secreting B cells in 100% (4/4) of KTRs (median 618, range 172–1,000 RBD-specific IgG-secreting B cells/10^6^ PBMC). We observed a strong correlation of detected numbers of RBD-specific IgG-secreting B cells and SARS-CoV-2-specific NAb titers (*r_s_* = 0.9040 and *P* < 0.0001) of KTRs (**Figure 3D**).

### 3.7 Serum neutralization capacities for Omicron BA.5 are higher in heterologously vaccinated kidney transplant recipients

We infected Vero-E6 cells with SARS-CoV-2 Omicron sublineage BA.5 to determine the serum neutralization capacity of KTRs for the currently dominant SARS-CoV-2 variant in Germany (34). We detected significantly higher rates of KTRs with infection neutralization for Omicron BA.5 in heVac [75.0% (6/8)] compared to hoVac [22.2% (4/18)] KTRs after second (*P* = 0.0256) and third heVac 100% (8/8); hoVac: 55.6% (10/18) (*P* = 0.0308) vaccination, respectively (**Figure 3E**). Serum neutralization capacities for Omicron BA.5 significantly increased in hoVac (*P* = 0.0078) and heVac (*P* = 0.0078) KTRs after third vaccination, respectively (**Figure 3F**).

### 3.8 Strong correlation of serum neutralization capacity for Omicron BA.5 with severe acute respiratory syndrome coronavirus 2–specific neutralizing antibodies and numbers of interleukin 2–secreting T cells

Spearman correlation analysis revealed a strong correlation of SARS-CoV-2-specific NAb titers of hoVac and heVac KTRs with the respective serum neutralization capacities for Omicron BA.5 (*r_s_* = 0.9045 and *r_s_* = 0.8959, *P* < 0.0001, respectively) (**Figure 3G**). For numbers of spike S1- and S2-specific IL-2 secreting cells of hoVac and heVac KTRs, we observed a strong correlation with the respective serum neutralization capacities for Omicron BA.5 (**Figures 3H** and Additional file 3: **Figure S3**). Spike S1- and S2-reactive IFN-γ and bifunctional IFN-γ/IL-2-secreting cells of hoVac and heVac KTRs correlated moderately to strongly with the respective serum neutralization capacities for Omicron BA.5 (**Figure 3H** and Additional file 3: **Figure S3**).

### 3.9 Severe acute respiratory syndrome coronavirus 2–specific T-cell responses of coronavirus disease 2019−vaccinated kidney transplant recipients strongly correlate with neutralizing antibody titers after two but not after three vaccinations

**Figure 4:**
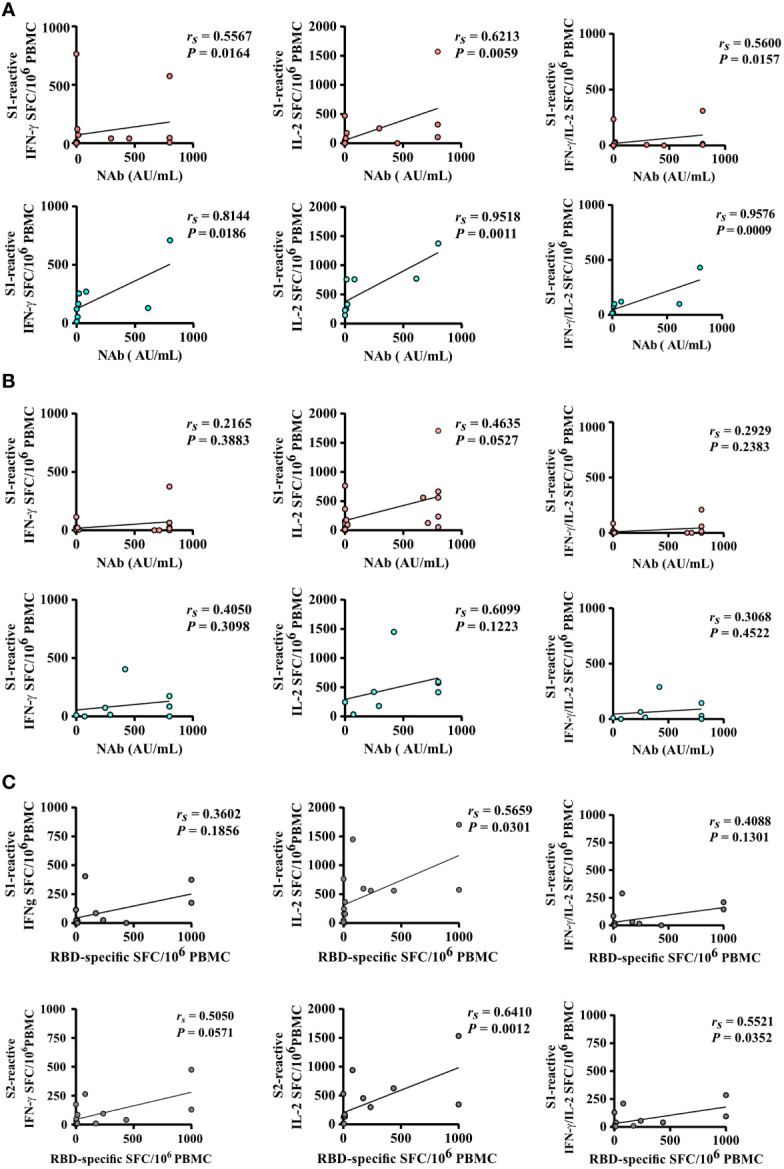
Correlation of SARS-CoV-2-specific T- and B-cell responses after two and three doses of COVID-19 vaccines. **(A, B)** Correlation of the numbers of spike S1-reactive IFN-γ, IL-2, and bifunctional IFN-γ/IL-2-secreting cells (depicted as SFCs per 10^6^ PBMC) of homologously (hoVac) (pink) or heterologously (heVac) (turquoise) vaccinated KTRs with SARS-CoV-2-specific NAb titers after two **(A)** or three **(B)** doses of COVID-19 vaccines. **(C)** Correlation of the numbers of spike (S1 or S2)-reactive IFN-γ, IL-2, and bifunctional IFN-γ/IL-2 SFC per 10^6^ PBMC with the numbers of RBD-specific memory B cells of hoVac and heVac KTRs after three doses of COVID-19 vaccines. Statistical analyses by correlation and linear regression; *r_s_* denotes the Spearman correlation coefficient.

Spearman correlation analysis revealed a strong correlation of numbers of spike S1-reactive IFN-γ, IL-2, and bi-functional IFN-γ/IL-2 secreting cells and SARS-CoV-2-specific NAb titers after the second vaccination of hoVac (*r_s_* = 0.5567, *r_s_* = 0.6213, and *r_s_* = 0.5600, respectively) and heVac (*r_s_* = 0.8144, *r_s_* = 0.9518, and *r_s_* = 0.9576, respectively) KTRs, respectively (**Figure 4A**). S2-reactive T cells did strongly correlate for IL-2-secreting cells and SARS-CoV-2-specific NAb titers (*r_s_* = 0.6316) in hoVac KTRs after two vaccinations (Additional file 4: **Figure S4A**). For two-times heVac KTRs, we observed a strong correlation of S2-reactive IFN-γ, IL-2, and bifunctional IFN-γ/IL-2- secreting cells and SARS-CoV-2-specific NAb titers (*r_s_* = 0.7904, *r_s_* = 0.9341, and *r_s_* = 0.7892, respectively), which was significantly less pronounced after three vaccinations (**Figure 4B** and Additional file 4: **Figure S4B**).

We further performed Spearman correlation analyses between SARS-CoV-2-specific T-cell responses and the numbers of RBD-specific IgG-secreting memory B cells upon three COVID-19 vaccinations in KTRs. Here we detected a moderate-to-strong correlation of spike S1- and S2-reactive IFN-γ, IL-2, and bifunctional IFN-γ/IL-2-secreting cells and numbers of RBD-specific IgG- secreting memory B cells (**Figure 4C**).

### 3.10 Significantly lower severe acute respiratory syndrome coronavirus 2–specific T-cell responses in severe acute respiratory syndrome coronavirus 2–specific neutralizing antibody–negative kidney transplant recipients after second but not after third coronavirus disease 2019 vaccination

**Figure 5:**
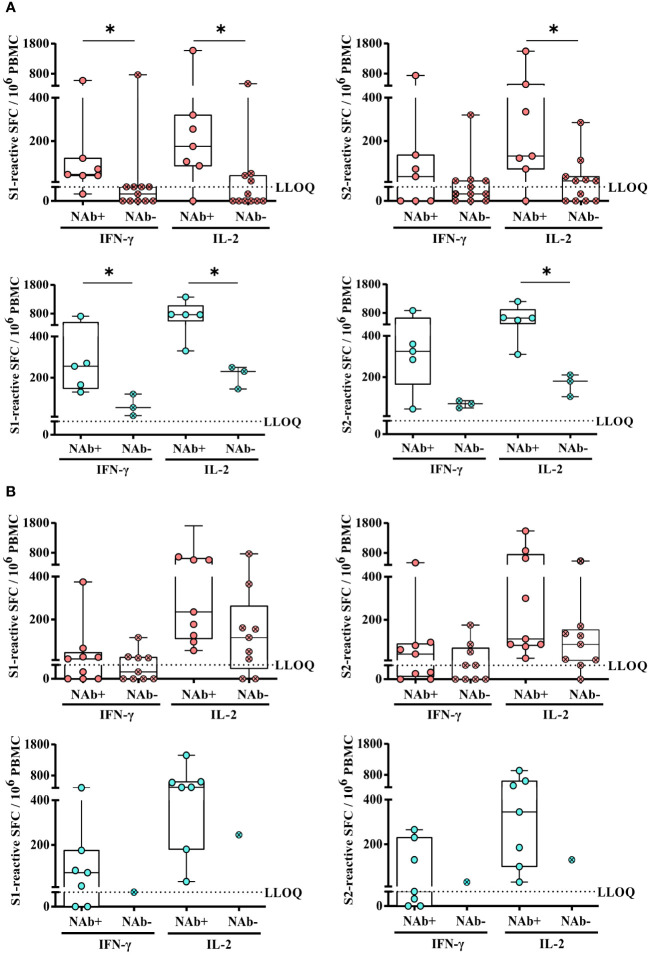
Comparison of SARS-CoV-2-specific T-cell responses of homologously or heterologously vaccinated KTRs with or without SARS-CoV-2-specific NAbs upon second or third COVID-19 vaccination. **(A)** and **(B)** Numbers of spike (S1 or S2)-reactive IFN-γ- and IL-2- secreting cells (depicted as SFCs per 10^6^ PBMC) of homologously (pink) or heterologously (turquoise) vaccinated KTRs with (pink and turquoise; open circle) or without (pink and turquoise, circle/cross) SARS-CoV-2-specific NAbs after second **(A)** or third vaccination **(B)**. Statistical analyses by two-sided Mann–Whitney U tests. Solely significant differences are indicated with an asterisk in the graphs. *P < 0.05. LLOQ, lower limit of quantification.

Next, we compared the magnitude of SARS-CoV-2-specific T-cell responses in KTRs with or without SARS-CoV-2-specific NAbs upon second or third vaccination. Indeed, upon second vaccination, we detected significantly lower numbers of spike S1-reactive IFN-γ, IL-2, and bifunctional IFN-γ/IL-2-secreting cells in hoVac (*P* = 0.0102, *P* = 0.0172, and *P* = 0.0090, respectively) and heVac (*P* = 0.0357, *P* = 0.0179, and *P* = 0.0179, respectively) NAb-negative KTRs, respectively (**Figure 5A** and Additional file 5: **Figure S5A**). In terms of S2-reactive cells, we observed significantly lower numbers of IL-2 but not IFN-γ and bifunctional IFN-γ/IL-2-secreting cells in hoVac (*P* = 0.0179) and heVac (*P* = 0.0357) NAb-negative KTRs (**Figure 5A** and Additional file 5: **Figure S5A**). However, these differences in T-cell reactivity were no longer detectable after third vaccination (**Figure 5B** and Additional file 5: **Figure S5B**).

### 3.11 Severe acute respiratory syndrome coronavirus 2 breakthrough infections

A SARS-CoV-2 breakthrough infection indicated by a positive SARS-CoV-2 PCR result occurred in 38.5% (10/26) of KTRs. This corresponds to 37.5% (3/8) of heVac and 38.9% (7/18) of hoVac KTRs. In seven (70.0%) of these KTRs, the breakthrough infection occurred after third vaccination, two (20.0%) KTRs had an infection after four, COVID-19 vaccinations and one (10.0%) after six COVID-19 vaccinations. In these KTRs, the median time between the SARS-CoV-2 infection and the last vaccination was 119 days (56–263 days). There were 90% of the infected KTRs who had a mild course of COVID-19 receiving outpatient care. One KTR was hospitalized with a moderate course of COVID-19 without any need for oxygen therapy and intensive care, but immunosuppressive therapy was reduced for 10 days and the patient was treated with SARS-CoV-2-specific monoclonal antibodies (sotrovimab) and remdesivir. Another infected KTR was also treated with SARS-CoV-2-specific monoclonal antibodies (sotrovimab). None of the KTRs reported recurrent SARS-CoV-2 infections or suspicions of long Covid (symptoms longer than 3 months after infection) (35).

**Figure 6:**
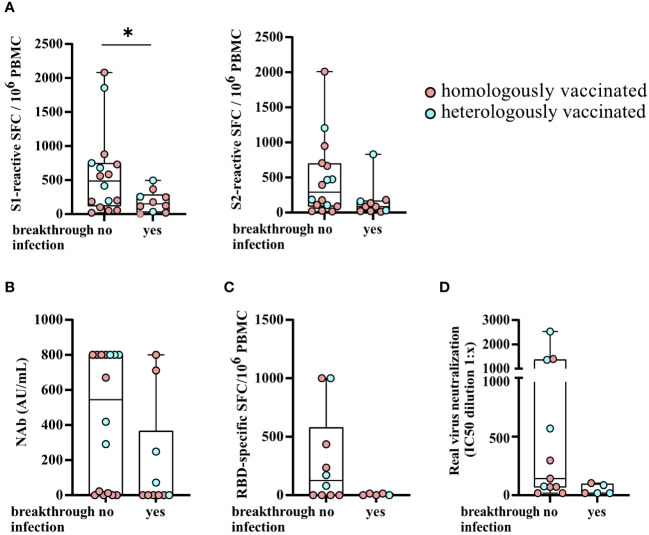
SARS-CoV-2-specific T- and B-cell responses and serum neutralization capacities for Omicron BA.5 stratified by KTRs with a SARS-CoV-2 breakthrough infection after third vaccination. **(A)** Numbers of SARS-CoV-2 spike S1- and S2-reactive T cells (depicted as the SFCs per 10^6^ PBMCs), SARS-CoV-2-specific NAb titers (AU/ml) **(B)**, the numbers of RBD-specific IgG- secreting memory B cells **(C)**, and serum neutralization capacities for Omicron BA.5 **(D)** stratified by KTRs with a SARS-CoV-2 breakthrough infection after third vaccination. Statistical analyses by two-sided Mann–Whitney U tests. Solely significant differences are indicated with an asterisk in the graphs. *P < 0.05.

KTRs with a SARS-CoV-2 breakthrough infection had significantly lower numbers of S1-reactive (*P* = 0.0478) and, by trend, lower numbers of S2-reactive T cells (*P* = 0.0585) and SARS-CoV-2-specific NAb titers (*P* = 0.0595) after third vaccination (**Figures 6A, B**). There were no significant differences between the two groups (KTRs with or without a breakthrough infection) with respect to age, sex, Charlson Comorbidity Index, eGFR, CRP, WBC, lymphocyte counts, CMV and BK viremia, and the type of immunosuppression (data not shown) and the numbers of RBD-specific IgG- secreting memory B cells (**Figure 6C**) and serum neutralization capacity for Omicron BA.5 (**Figure 6D**).

## 4 Discussion

KTRs receiving immunosuppressive therapy are at higher risk for poor COVID-19-related outcomes and therefore in urgent need for establishing a vaccine-induced SARS-CoV-2-specific humoral and cellular immune response to prevent severe COVID-19 (15, 36–38). Immunosuppressive therapies could hinder (19, 37) and the administration of different vaccine regimens could influence the development of vaccine-induced immune responses in KTRs. As a consequence, close vaccine–accompanying immune monitoring is highly recommended for KTRs to assess the vaccination effectiveness (38, 39).

The major goal of this study was to perform a comparative in-depth analysis of SARS-CoV-2-specific cellular and humoral immune responses in hoVac and heVac KTRs to allow for a broader insight into vaccine-induced immunity in this patient cohort using different COVID-19 vaccination regimens.

This study had several important findings. (i) Upon third vaccination, hoVac and heVac KTRs showed similar T-cell response rates (100% in both cohorts) and comparable numbers of SARS-CoV-2-reactive T cells. (ii) The cytokine expression pattern (proportion of mono- and bifunctional cells) of the detected SARS-CoV-2-reactive T cells was comparable between hoVac and heVac KTRs after second and third vaccination. Monofunctional SARS-CoV-2-reactive T cells showed a dominance of IL-2 secretion at both time points. (iii) Moreover, the cytokine production of SARS-CoV-2-reactive T cells per single cell was similar between both subcohorts after second and third vaccination. (iv) SARS-CoV-2-specific NAb titers were comparable between hoVac and heVac KTRs, whereas NAb positivity rates were significantly higher in heVac KTRs upon third vaccination. (v) SARS-CoV-2-specific T-cell responses of hoVac and heVac KTRs strongly correlated with SARS-CoV-2-specific NAb titers after two vaccinations. (vi) HeVac KTRs showed significantly higher serum neutralization capacities for Omicron BA.5. (vii) SARS-CoV-2 breakthrough infections after third vaccination occurred in equal numbers in hoVac and heVac KTRs.

The utilization of a highly robust and therefore highly suitable multiplex Fluorospot assay for longitudinal studies allowed for the quantification of the numbers of SARS-CoV-2-reactive IFN-γ- and/or IL-2 secreting cells, the distinct cytokine expression pattern of reactive cells, and the amount of IFN-γ and/or IL-2 production per reactive cell.

In this study, heVac KTRs showed overall higher spike-reactive T-cell response rates and significantly higher numbers of spike S1- and S2-reactive IFN-γ, IL-2, and bifunctional IFN-γ/IL-2- secreting cells compared to hoVac KTRs after receiving two doses of COVID-19 vaccination, which is consistent with previous reports in immunocompetent individuals (40, 41). Sattler et al. reported spike-specific CD4 T-cell responses in more than 90% of two-times hoVac (2x BNT162b2 mRNA vaccine) KTRs (38), which is a higher proportion as compared to our results (77.8%) of hoVac KTRs. The Sattler study and ours differ in the time point of T-cell analysis after vaccination as well as the used assays, which could result in the observed slight differences, whereas the patient characteristics regarding age and sex were comparable between both studies. Upon third vaccination, the numbers of SARS-CoV-2-reactive T cells in hoVac KTRs increased significantly, resulting in comparable numbers of SARS-CoV-2-reactive T cells as well as overall T-cell response rates between the hoVac and heVac KTRs of our study. In line with this, Thompson et al. reported comparable numbers of spike-reactive T cells between hoVac and heVac KTRs after four vaccinations (17).

Most notably, 100% of KTRs included in this study were able to develop SARS-CoV-2-specific T-cell responses after a third COVID-19 vaccine dose, regardless of the administration of homologous or heterologous vaccine regimens. This is in contrast to studies of Bertrand et al. (18) and Heinzel et al. (19) who detected SARS-CoV-2-specific T-cell responses only in 70% and 89% of three times hoVac (mRNA vaccine) KTRs, which might be due to using assays limited to the detection of IFN-γ-related T-cell responses. The baseline patient characteristics in both mentioned studies were comparable to our study, whereas immunosuppressive regimens differed in the study of Heinzel et al. (19). The study of Heinzel et al. (19) also included heVac KTRs, who also showed lower T-cell response rates compared to the heVac KTRs of our study, but vaccination schemes were different with two mRNA vaccine doses followed by a third vaccination with a vector vaccine that limits the comparability to our results.

Our important finding of SARS-CoV-2-specific T-cell response rates in 100% of KTRs upon third vaccination in both subcohorts might indicate that the diminished COVID-19 vaccine–induced T-cell reactivity upon homologous vaccination was overcome after three vaccinations. Since SARS-CoV-2-specific T-cell responses are associated with milder courses of COVID-19 (42–44) and might form an additional correlate of protection to SARS-CoV-2-specific antibodies against severe COVID-19 (45, 46), this could probably result in less severe COVID-19 after breakthrough infections in three times or manifold vaccinated KTRs. Furthermore, there are reports showing that despite reduced neutralizing activity against the variants of concern, the majority of T-cell responses are preserved in both the vaccination and natural infection setting and therefore might be effective in controlling infections with VOC (10, 47–53) also in KTRs. Overall, in line with other reports, this demonstrates the importance of continuous COVID-19 vaccinations in KTRs (38, 54).

Despite the different observed kinetics of SARS-CoV-2-specific T-cell responses between hoVac and heVac KTRs, the proportions of SARS-CoV-2-reactive mono- and bifunctional cells were comparable between both cohorts at both time points. After two vaccinations, we observed a predominantly monofunctional spike-specific T-cell response in both cohorts with IL-2-secreting cells being more frequently compared to IFN-γ-secreting cells. This observation is in contrast to the findings of others (38, 55), who detected only low frequencies of IL-2-producing (CD4) T cells in dual-vaccinated KTRs. Noteworthy, after third vaccination, the numbers of bifunctional spike S1- and S2-reactive T cells tended to be lower in both cohorts, accompanied by an increase of monofunctional cells, showing a more pronounced dominance of IL-2 secretion with only very limited proportions of monofunctional IFN-γ-secreting cells. The latter was already observable in heVac KTRs after second vaccination. In contrast to the shift toward a more monofunctional expression profile of SARS-CoV-2-reactive T cells, the amount of produced IFN-γ and IL-2 per single reactive cell was higher in bifunctional cells. This is in line with a previous report on SARS-CoV-2 infected individuals, which also detected a higher relative cytokine production in poly- compared to monofunctional cells (56). Considering the importance of polyfunctional cells for controlling viral infections (42, 57–59), those bifunctional T cells could be important when it comes to SARS-CoV-2 infections in KTRs. The cytokine expression pattern (higher amounts of IL-2-secreting cells) of the detected SARS-CoV-2-reactive T cells in our cohort indicate a CD4 T-cell phenotype (12) and importantly argues against an absent or defective CD4 T-cell help for neutralizing antibody production (11, 60) of B cells in this cohort.

Previous studies that explored cellular SARS-CoV-2-specific immunity in KTRs mainly focused on assays predominantly analyzing IFN-γ-related immune responses (17, 18, 61). This approach might lead to an underestimation of the SARS-CoV-2-specific T-cell immunity in KTRs, when, e.g., IL-2-related immune responses are not detected (62). This has to be considered to be particularly important since it is known that COVID-19 vaccine–induced T-cell responses do not rapidly decline as virus-specific and virus-neutralizing antibodies (45, 63).

SARS-CoV-2-specific NAbs are known to prevent and contain infection (64–66) and are regarded together with anti-spike IgG concentration as the correlates of protection for vaccines against symptomatic COVID-19 (67). Routine testing for antiviral vaccine-induced immunity in KTRs is often limited to serological tests identifying anti-SARS-CoV-2 IgG and, to a lesser extent, SARS-CoV-2-specific NAbs, which are both frequently absent in KTRs also after third vaccination (14–16). This is in accordance with the results of our study, where only 42.3% and 61.5% of KTRs developed SARS-CoV-2-specific NAbs upon two or three COVID-19 vaccine doses, respectively. Especially, hoVac KTRs showed only very limited capacities of developing SARS-CoV-2-specific NAbs after two and three vaccine doses (33.3% and 50.0%, respectively). However, as we were able to demonstrate that all KTRs in our cohort developed T-cell responses upon third vaccination—probably in KTRs who do not develop NAb responses—a more differentiated workup of the vaccination response seems to be advisable where possible. It appears that NAb negativity alone might not be a sufficient parameter to stop COVID-19 vaccinations in an individual KTR as this might be not sufficient to declare a KTR a COVID-19 vaccine non-responder. Moreover, as indicated by others (61), a short-term withdrawal of MMF to induce seroconversion upon COVID-19 vaccination should be evaluated critically and NAb negativity alone might not be a sufficient parameter to make such a decision.

In addition to the low SARS-CoV-2-specific NAb-positivity rates, our study results demonstrate that approximately one-third of KTRs who tested negative for NAbs showed RBD-specific IgG-secreting B cells in ELISpot analysis. The finding of a strong correlation of detected numbers of RBD-specific IgG-secreting B cells and SARS-CoV-2-specific NAb titers of KTRs indicate that IgG secreted by RBD-specific memory B cells show neutralizing activity. Those existing SARS-CoV-2-specific memory B cells in some of the KTRs who tested negative for SARS-CoV-2-specific NAb might be hindered in producing NAbs in the *in vivo* setting, maybe as a consequence of immunosuppression.

Whereas a third COVID-19 vaccination resulted in a significant increase in SARS-CoV-2-specific NAb titers in both cohorts, the magnitudes of SARS-CoV-2-specific NAb titers were comparable between hoVac and heVac KTRs at both time points. This is in contrast to other studies, which reported lower SARS-CoV-2-specific NAb titers in homologously vaccinated immunocompetent individuals (40, 41, 68, 69).

Clinical routine testing for antiviral vaccine-induced immunity includes NAb or anti-S antibody detection against the original SARS-CoV-2 strains and not against VOC. To test whether vaccination with original COVID-19 vaccines offers effective protection toward VOC Omicron, we determined the serum neutralization capacities of KTRs after two and three COVID-19 vaccinations for Omicron BA.5, which is the currently dominant SARS-CoV-2 variant in Germany (34). After third vaccination, all heVac but approximately half of the hoVac KTRs showed serum neutralization capacities for Omicron BA.5. Interestingly, SARS-CoV-2-specific NAb titers and the numbers of spike-reactive IL-2 secreting cells after three vaccinations strongly correlated with serum neutralization capacities for Omicron BA.5. Since the serum neutralization capacities for Omicron BA.5 significantly increased in both cohorts, further COVID-19 vaccination doses might be beneficial for KTRs to broaden immunity against other emerging variants as it was seen by others in hemodialysis patients (25).

In compliance with previous reports, we identified a low eGFR in KTRs as an indicator for lacking SARS-CoV-2-specific NAb development after second and third COVID-19 vaccination (37, 54). Furthermore, KTRs who did not develop NAbs were under MMF-therapy unexceptional, at least following two doses of COVID-19 vaccines. Other studies also reported of weakened or negative antibody responses in solid organ transplant recipients under MMF therapy upon COVID-19 vaccination (16, 37, 70, 71). Interestingly, immunosuppressive treatment did not seem to inhibit the development of COVID-19 vaccine-induced T-cell responses in principle in KTRs upon second and third vaccination, although MMF is known to hamper the synthesis of the guanine-carrying nucleotide guanosine and consequently the proliferation of B and T lymphocytes (72).

In this study, 10 KTRs showed a SARS-CoV-2 breakthrough infection with a predominantly mild course of COVID-19, which is in line with others reporting of predominantly mild courses of COVID-19 in three-times vaccinated healthy subjects (11, 42, 73, 74) and KTRs (54, 61). Interestingly, we observed evenly distributed cases of breakthrough infections in hoVac compared to heVac KTRs and KTRs with a breakthrough infection had significantly lower spike S1-specific T-cell responses as compared to uninfected KTRs. This is in line with the observations of others (46).

This study has limitations. Foremost, there is a relatively small sample size, especially in the heVac group. We focused on including a well-characterized cohort of hoVac and heVac KTRs, with comparable patient characteristics and clinical parameters, which limited the number of subjects that could be included. Further, we do not have data on the long-term development of COVID-19 vaccine–induced cellular and humoral immune responses in our cohort of KTRs. Therefore, future studies allowing an evaluation of the persistence of the vaccine-induced responses in KTRs are urgently needed (45).

Overall, our findings bring important new insights into the vaccine-induced SARS-CoV-2-specific immunity in KTRs. Knowing the individual SARS-CoV-2-specific T-cell immunity of KTRs could reflect another key marker in preventive and protective immunity against at least severe causes of COVID-19 in immunocompromised patients (38, 75). Based on the findings of this study, it seems to be recommendable to not only assess humoral but also consider cellular SARS-CoV-2-specific immunity in KTRs to provide a comprehensive understanding of the COVID-19 vaccine–induced immune response in a transplant setting.

## Data availability statement

The raw data supporting the conclusions of this article will be made available by the authors, without undue reservation.

## Ethics statement

The study was approved by the Medical Ethics Committee of the Klinikum Rechts der Isar of the Technical University Munich (approval number 163/21 S-SR, March 19th, 2021) and registered at the Paul Ehrlich Institute (NIS592). The patients/participants provided their written informed consent to participate in this study.

## Author contributions

The authorship order among co-first authors was based on NK and CH-L having designed all experiments and outlined and wrote the manuscript. GW performed most of the experiments and helped writing the manuscript. MW, MT, and VL were responsible for data curation and created the database in its current form. B-HL and C-CC performed the infection-neutralization assays. NK, CH-L, UP, MB, TB, VK, HM, CC, LP, and LR conceptualized the project. NK, CH-L, CC, and GW were responsible for methodology. NK, CH-L, GW, and TB performed the investigations and analyzed the data. UP, MB, TB, UH, CS, and LR supervised the project. NK, CH-L, and GW wrote the original draft. NK, CH-L, UP, MB, TB, VA, and LR gave substantial input to the final version of the manuscript and reviewed and edited the manuscript with feedback from all authors. All authors have read and agreed to the published version of the manuscript.
