# Supplementary material for: Comparable cellular and humoral immunity upon homologous and heterologous COVID-19 vaccination regimens in kidney transplant recipients
Source: Front Immunol. 2023 Mar 31;14:1172477. doi: 10.3389/fimmu.2023.1172477 (PMC10102365; doi:10.3389/fimmu.2023.1172477)
Supplement: Supplementary file 1 [file DataSheet_1.pdf]

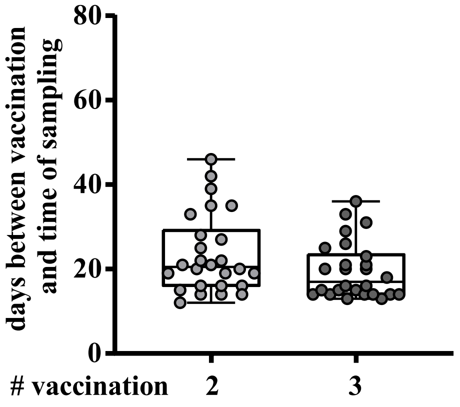

Additional file 1 Figure S1. Boxplot of the time interval in days between the second (left, light grey) and third (right, dark grey) vaccination and time of sampling. Dots represent single data points of patients. The boxes indicate the median, and 25th and 75th percentile of values. The whiskers show the minimum to maximum values.
