# Supplementary material for: Comparable cellular and humoral immunity upon homologous and heterologous COVID-19 vaccination regimens in kidney transplant recipients
Source: Front Immunol. 2023 Mar 31;14:1172477. doi: 10.3389/fimmu.2023.1172477 (PMC10102365; doi:10.3389/fimmu.2023.1172477)
Supplement: Supplementary file 2 [file DataSheet_2.pdf]

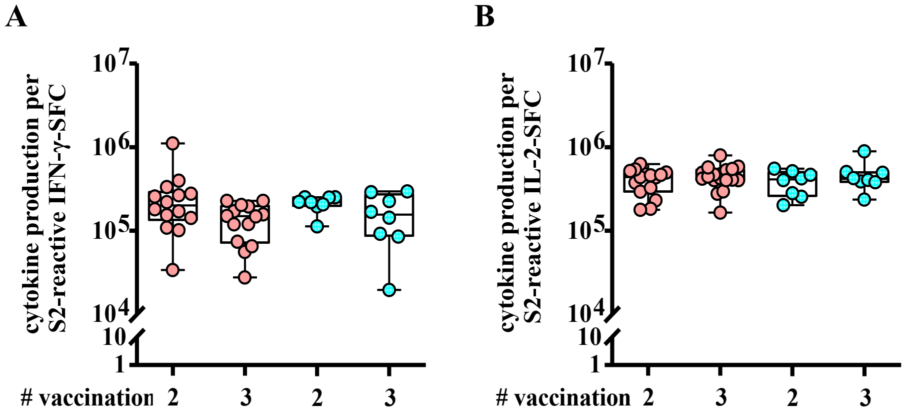

Additional file 2 Figure S2. Quantity of cytokine production of SARS-CoV-2 spike S2-reactive T cells of homologously and heterologously vaccinated KTR after two and three COVID-19 vaccinations. Comparison of IFN-γ **(A)** and IL-2 **(B)** production per spike S2-reactive cytokine secreting cell between homologously (pink) or heterologously (turquoise) vaccinated KTR after two or three doses of COVID-19 vaccines. Cytokine production per cell was determined by the parameters spot size and spot intensity as described in methods. Statistical analyses by two-sided Mann-Whitney tests. Solely significant differences are indicated with asterisk in the graphs.
