# Supplementary material for: Comparable cellular and humoral immunity upon homologous and heterologous COVID-19 vaccination regimens in kidney transplant recipients
Source: Front Immunol. 2023 Mar 31;14:1172477. doi: 10.3389/fimmu.2023.1172477 (PMC10102365; doi:10.3389/fimmu.2023.1172477)
Supplement: Supplementary file 3 [file DataSheet_3.pdf]

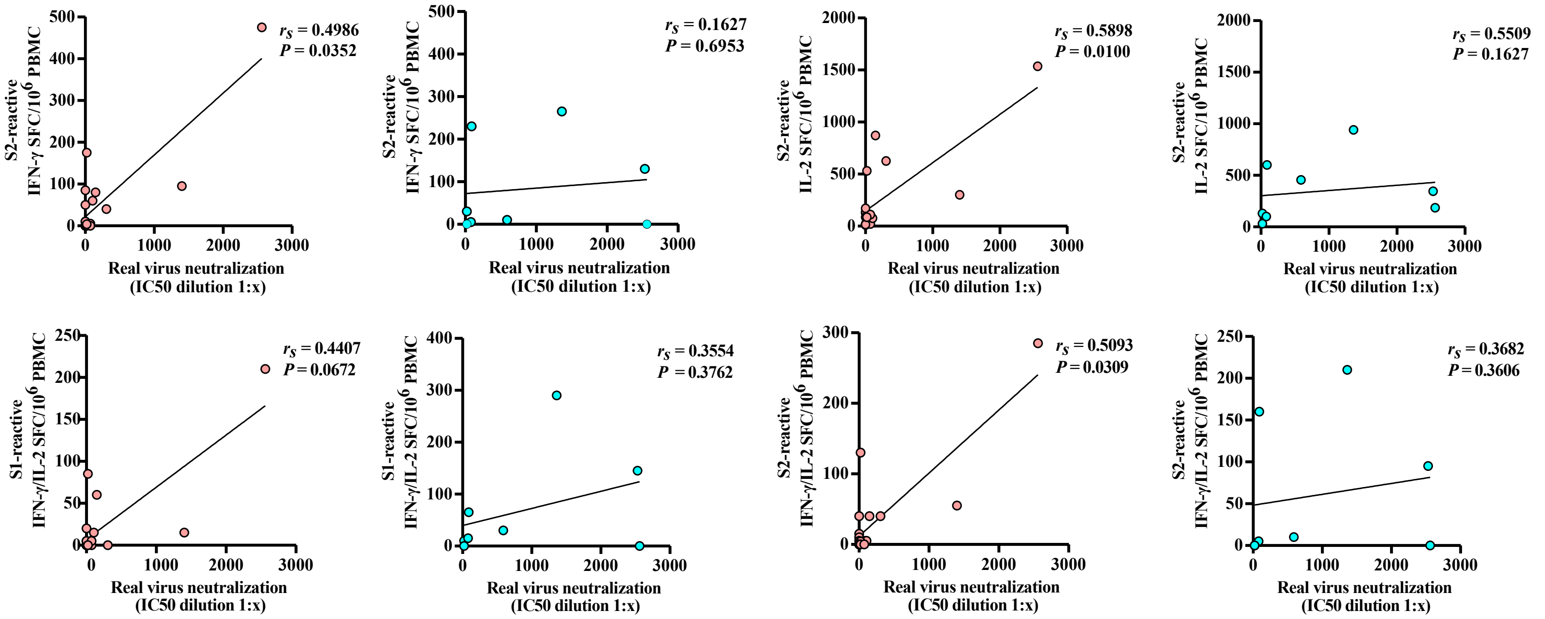


**Additional file 3 Figure S3. Spearman correlation of serum neutralization capacity for Omicron BA.5 and numbers of spike S1- and S2-specific IFN-γ, IL-2, and IFN-γ/IL-2 secreting cells.** Spearman correlation of serum neutralization capacities for Omicron BA.5 and numbers of spike S1- and S2-specific IFN-γ, IL-2, and bi-functional IFN-γ/IL-2 secreting cells depicted as spot-forming cells/SFC per $10^6$ PBMC after third vaccination of homologously and heterologously vaccinated KTR. $r_s$ denotes Spearman correlation coefficient.
