# Supplementary material for: Comparable cellular and humoral immunity upon homologous and heterologous COVID-19 vaccination regimens in kidney transplant recipients
Source: Front Immunol. 2023 Mar 31;14:1172477. doi: 10.3389/fimmu.2023.1172477 (PMC10102365; doi:10.3389/fimmu.2023.1172477)
Supplement: Supplementary file 4 [file DataSheet_4.pdf]

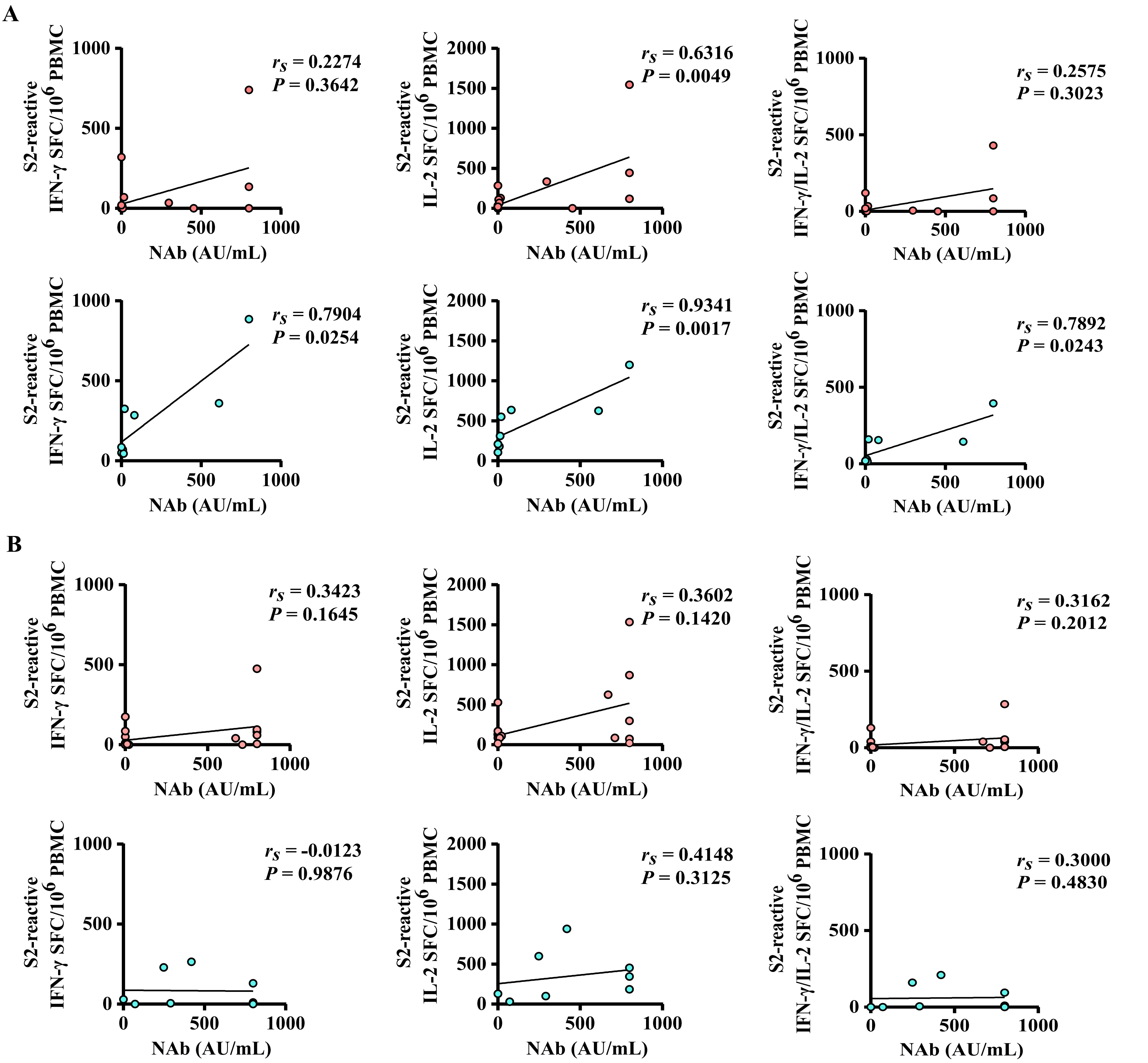

Additional file 3 Figure S3. Correlation of SARS-CoV-2 spike S2-specific T- and B-cell responses after two and three doses of COVID-19 vaccines. (A) and **(B)** Correlation of numbers of spike S2-reactive IFN-γ, IL-2, and bi-functional IFN-γ/IL-2 secreting cells (depicted as spot-forming cells/SFC per 10^6 PBMC) of homologously (pink) and heterologously (turquoise) vaccinated KTR with SARS-CoV-2-specific NAb titers after two (A) or three (B) doses of COVID-19 vaccines. Statistical analyses by correlation and linear regression; r_s denotes Spearman correlation coefficient.
