# Supplementary material for: Comparable cellular and humoral immunity upon homologous and heterologous COVID-19 vaccination regimens in kidney transplant recipients
Source: Front Immunol. 2023 Mar 31;14:1172477. doi: 10.3389/fimmu.2023.1172477 (PMC10102365; doi:10.3389/fimmu.2023.1172477)
Supplement: Supplementary file 5 [file DataSheet_5.pdf]

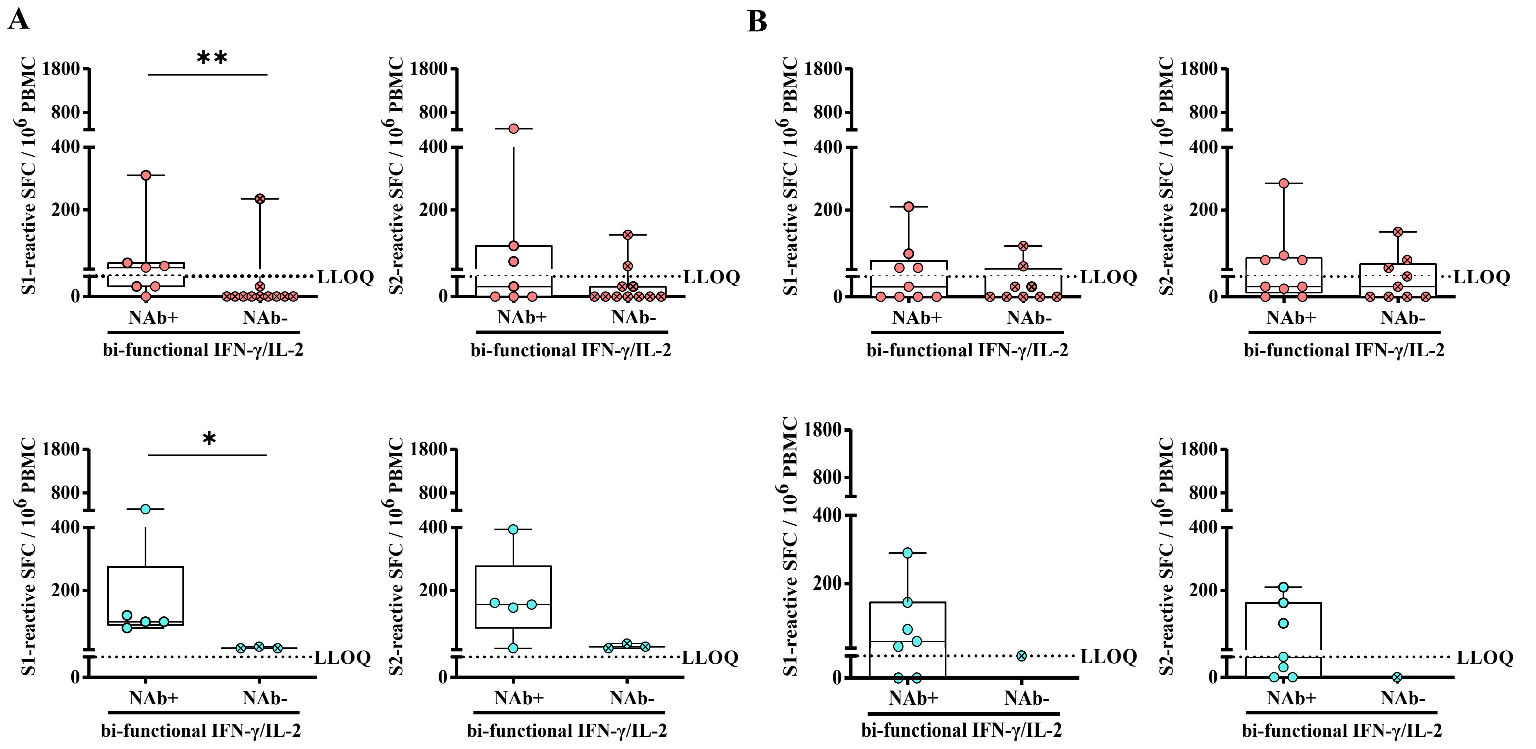

Additional file 5 Figure S5. Comparison of SARS-CoV-2-specific T-cell responses of homologously and heterologously vaccinated KTR with or without SARS-CoV-2-specific NAb titers after second or third COVID-19 vaccination. (A) and **(B)** Numbers of spike- (S1 or S2) reactive bi-functional IFN-γ/IL-2 secreting cells (depicted as spot-forming cells/SFC per 10^6 PBMC) of homologously (pink) or heterologously (turquoise) vaccinated KTR with (pink and turquoise; open circle) or without (pink and turquoise, circle/cross) SARS-CoV-2-specific NAb titers after second (A) or third (B) vaccination. Statistical analyses by two-sided Mann–Whitney-U tests. Solely significant differences are indicated with asterisk in the graphs. $**P < 0.01$. LLOQ = Lower limit of quantification.on.
